# Supplementary material for: Skipping Exon-v6 from CD44v6-Containing Isoforms Influences Chemotherapy Response and Self-Renewal Capacity of Gastric Cancer Cells
Source: Cancers (Basel). 2020 Aug 22;12(9):2378. doi: 10.3390/cancers12092378 (PMC7564355; doi:10.3390/cancers12092378)
Supplement: Supplementary file 1 [file cancers-12-02378-s001.pdf]

Supplementary Material: Skipping Exon-v6 from CD44v6-Containing Isoforms Influences Chemotherapy Response and Self-Renewal Capacity of Gastric Cancer Cells

Silvana Lobo, Carla Pereira, Carla Oliveira and Gabriela M. Almeida

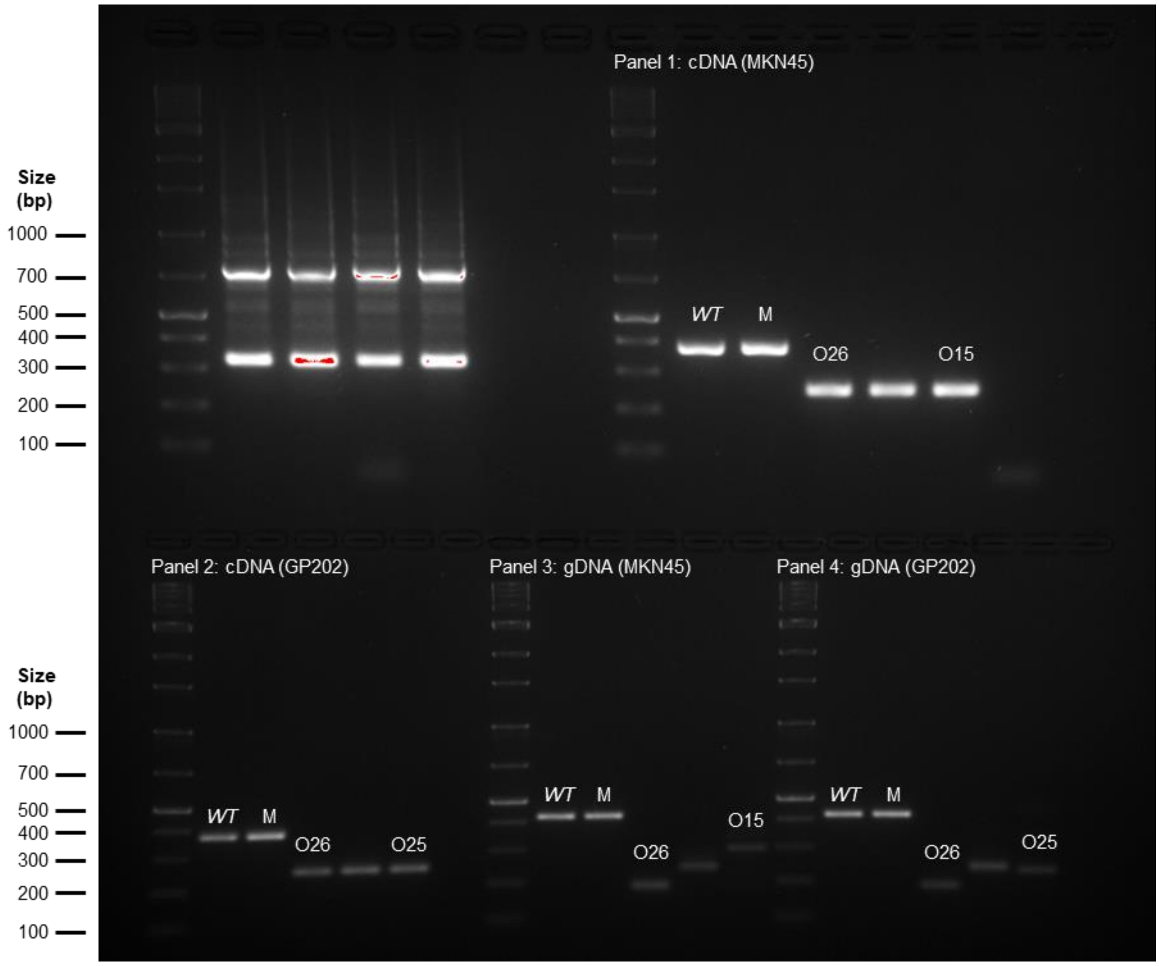

Figure S1. Uncropped image of the 2% agarose gel from Figure 2A.

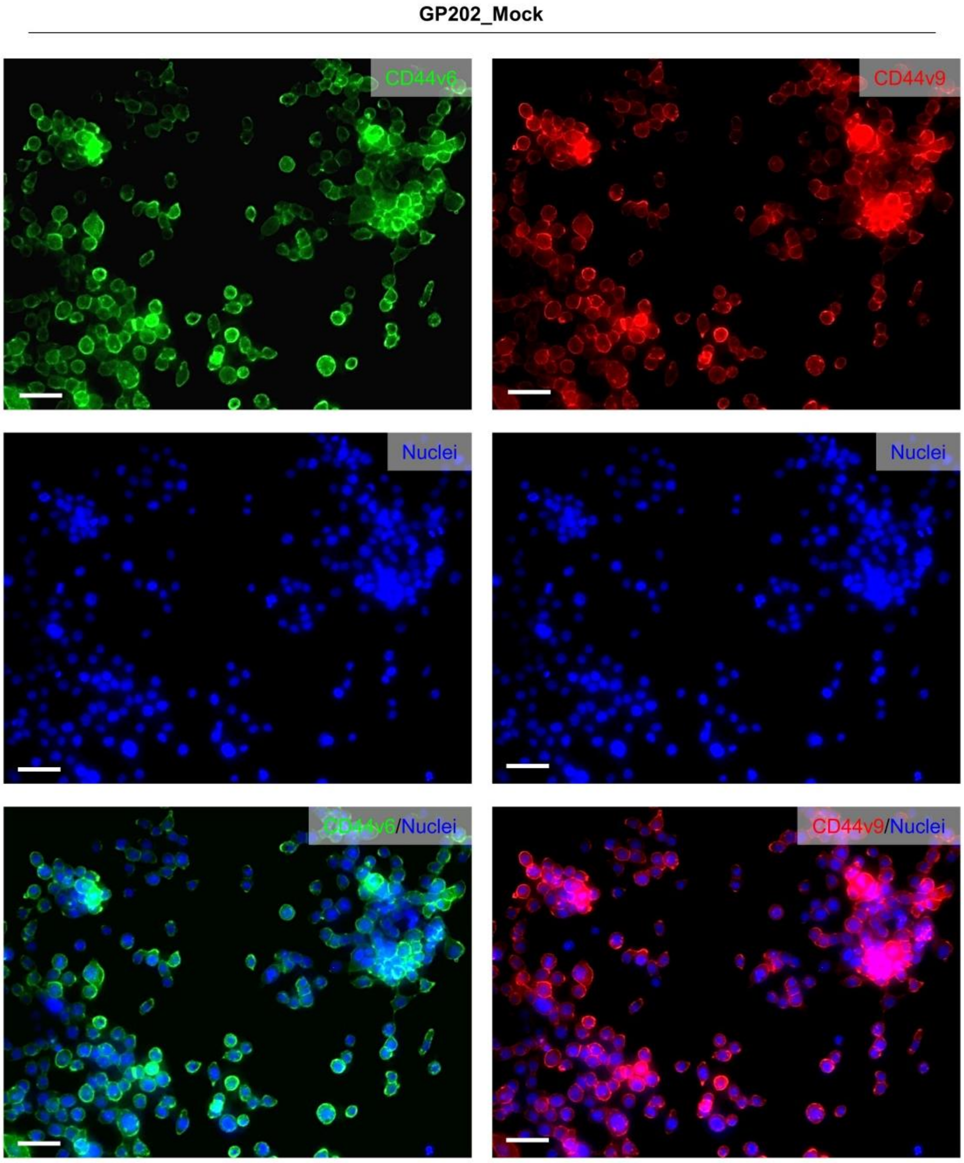

Figure S2. Illustrative immunofluorescence images highlighting CD44v6 (green) and CD44v9 (red) membranous staining observed in Figure 3C. Nuclei were stained with DAPI (blue) and white scale bars represent a distance of 50 µm.

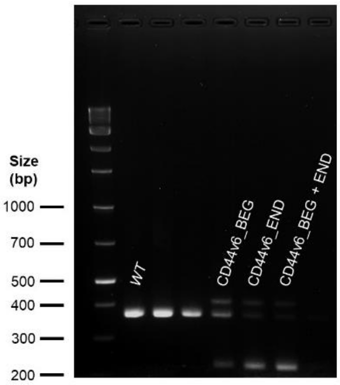

Figure S3. Uncropped image of the 2% agarose gel from Figure 6C.

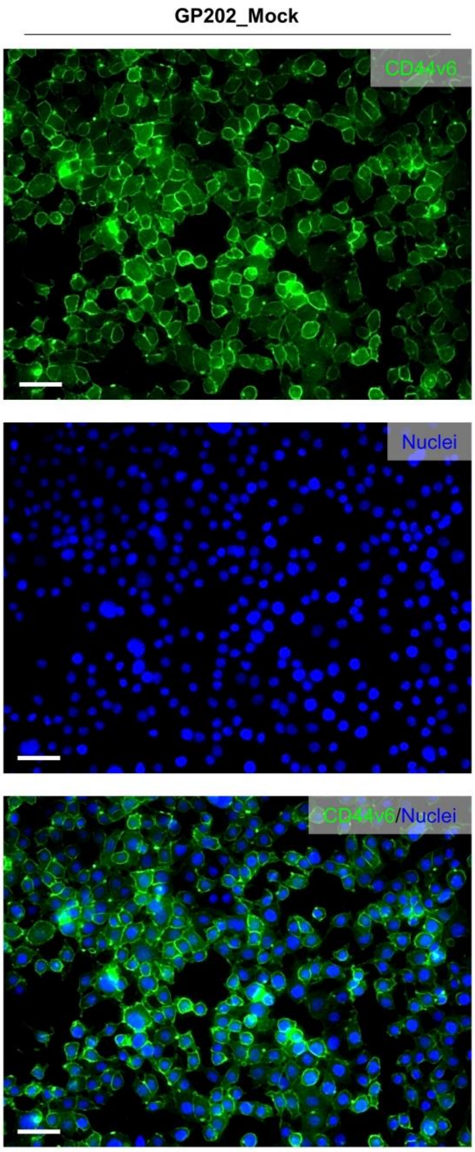

Figure 4. Illustrative immunofluorescence images highlighting CD44v6 (green) membranous staining observed in Figure 6E. Nuclei were stained with DAPI (blue) and white scale bars represent a distance of 50 μm.
